# Secondary Cooling Water System Control Method Based on Deep Reinforcement Learning

**DOI:** 10.3390/s26092783

**Published:** 2026-04-29

**Authors:** Jin Xu, Yu Cheng, Cheng Shen, Qingxin Zhang

**Affiliations:** School of Artificial Intelligence, Shenyang Aerospace University, Shenyang 110136, China; sh_xujin@126.com (J.X.); chengyu1@stu.sau.edu.cn (Y.C.);

**Keywords:** secondary cooling water system, deep reinforcement learning, proximal policy optimization (PPO), beta distribution, intelligent control, industrial process control

## Abstract

The secondary cooling water system is difficult to control because of loop coupling, thermal inertia, and strict actuator constraints. In addition, when conventional proximal policy optimization (PPO) uses Gaussian action sampling with clipping, the mismatch between sampled and executed actions may degrade learning and control smoothness near actuator limits. To address these issues, this paper develops a Beta-policy and PID-inspired augmented-state proximal policy optimization framework, termed BPAS-PPO, for the secondary cooling water system. The framework augments the state with proportional, integral, and derivative error features, adopts a Beta-distribution policy for bounded continuous-action generation, and uses a piecewise dense reward for the dual-loop tracking task. Simulation studies on an identified linear two-input two-output (TITO) model within the selected operating region show that the complete PID-augmented state yields the most effective training representation among the tested alternatives. Compared with PID, Fuzzy-PID, and Gauss-PPO, BPAS-PPO shows lower overshoot, shorter settling time, better setpoint tracking and disturbance rejection, and smoother control actions near actuator limits. The proposed framework is effective for the studied system within the selected operating region, while its performance beyond this region requires further validation.

## 1. Introduction

In industrial intelligent control, sensors and control methods are closely connected [1,2]. Sensors provide real-time measurements of system states and form the basis for control decisions. In turn, intelligent control algorithms can use these data to improve closed-loop control performance [3]. In secondary cooling water systems, key variables such as water temperature, pressure, and flow rate are usually monitored by industrial sensors in real time [4]. These measurements provide essential information for condition monitoring and control. However, because such systems often exhibit strong multivariable coupling, significant thermal inertia, and time-varying dynamics, improving measurement accuracy alone is not enough to achieve high-performance control under complex operating conditions. Therefore, it is important to develop advanced intelligent control methods for secondary cooling water systems.

The main goal of high-performance control in secondary cooling water systems is to deliver the required cooling capacity to the load side through regulation of supply water temperature and flow rate. Accurate regulation of these variables is essential for adapting to changing thermal conditions and maintaining efficient heat transfer. High-performance control also requires effective matching between cooling supply and time-varying thermal demand to ensure reliable and efficient operation. Fast dynamic response, strong disturbance rejection, and smooth actuator operation are additional performance requirements under varying operating conditions. These requirements are difficult to satisfy in practice because cooling capacity depends on the coordinated regulation of water temperature and flow rate, resulting in strong coupling between control variables. Moreover, thermal load fluctuations, environmental disturbances, and variations in equipment conditions alter the system dynamics over time, thereby challenging the effectiveness of conventional control methods under changing operating conditions.

Under these conditions, PID control has been widely used in industrial thermal systems because of its simple structure and easy implementation [5,6]. For example, Mei et al. proposed a fractional-order PID strategy for cooling water temperature control, which improved response speed and reduced overshoot [7]. However, fixed controller gains may no longer provide satisfactory performance when thermal loads vary significantly or heat transfer becomes strongly nonlinear. To improve adaptability, fuzzy PID control has been introduced [8,9]. Tunjung et al. developed an adaptive fuzzy PID controller for a water thermal mixing process and achieved better dynamic control performance [10]. Although fuzzy PID improves robustness to some extent, its rule design often depends on expert knowledge, and performance may degrade under wide-range operating conditions with strong coupling and time-varying dynamics. For multivariable systems with constraints, model predictive control (MPC) provides a more systematic solution [11,12,13]. Liu et al. combined genetic algorithms with MPC to jointly optimize pump frequency and supply temperature setpoints in HVAC chilled water systems, significantly reducing energy consumption while meeting cooling demand [14]. However, MPC relies on model accuracy, and continuously changing loads in secondary cooling systems often cause model mismatch that degrades performance. The online optimization burden further limits its applicability in fast real-time tasks. Heuristic optimization methods have also been applied [15,16,17]. Wang et al. combined differential evolution with load prediction to perform global predictive optimization for air-conditioning water systems in data centers, improving both energy efficiency and response delay [18]. These limitations collectively suggest that effective control of secondary cooling water systems requires an approach capable of handling loop coupling and time-varying dynamics with reduced reliance on highly accurate process models for online computation.

To address these limitations, deep reinforcement learning (DRL) has attracted increasing attention in complex process control. Unlike methods that require an explicit analytical process model for online computation, DRL learns control policies through interaction with a simulated or real environment, making it well suited for systems with strong coupling and significant inertia. Early value-based methods such as DQN demonstrated the feasibility of reinforcement learning in control tasks [19]. However, discrete action representations limit their direct applicability in industrial systems with continuous control inputs. To overcome this limitation, several DRL algorithms for continuous control have been developed and applied in energy systems and process control scenarios [20,21,22]. Although these methods have shown promising results, their practical application in industrial systems is still challenged by training instability, sensitivity to hyperparameter settings, and insufficient robustness under changing operating conditions. These challenges motivate the exploration of more stable and structured DRL algorithms for industrial process control.

Among DRL algorithms for continuous control, proximal policy optimization (PPO) has attracted increasing attention due to its clipped surrogate objective, which constrains policy updates and improves training stability [23,24,25,26]. In industrial control applications, Zhou et al. proposed a PPO-based controller for online PID gain tuning and incorporated error-difference information into the state representation [27]. Silva et al. further applied PPO to the control of conventional continuous thickeners and demonstrated its effectiveness in an industrial process with complex dynamics [28]. These studies indicate that PPO is a promising tool for industrial process control. However, when PPO is applied to secondary cooling water systems characterized by coupling, thermal inertia, and actuator constraints, two important issues remain insufficiently addressed.

First, the influence of different PID-inspired state representations on agent training has not been systematically investigated. Existing studies typically construct the state space from process variables and instantaneous tracking errors, with limited consideration of integral and derivative error features. For high-inertia thermal systems such as secondary cooling water processes, the current error alone is often insufficient to characterize system evolution under disturbances and varying operating conditions. As a result, the agent may fail to capture how the error evolves over time, which can impair both training convergence and closed-loop performance. Although some error-related variables have been considered in previous work, the impact of different PID-inspired error-feature combinations on policy learning remains unclear.

Second, a mismatch still exists between standard PPO action policies and the bounded actuator constraints in practical systems. In secondary cooling water systems, manipulated variables such as valve openings and pump frequencies are inherently limited by physical boundaries. However, PPO policies are commonly parameterized by Gaussian distributions, and sampled actions are then clipped or transformed to satisfy the feasible range. Such post-processing may distort the action distribution near the boundaries, leading to unstable exploration, action saturation, or abrupt control variations. Gu et al. addressed action boundary violations through soft constrained policy optimization [29], but this approach increases algorithmic complexity and online computational cost. Beta-distribution-based PPO has been proposed for continuous bounded action spaces and demonstrated promising performance in global path planning [30,31]. Because the Beta distribution is naturally defined on a finite interval, it is inherently compatible with bounded control actions and may help reduce the distribution distortion caused by Gaussian clipping. However, these studies have mainly focused on robotics and planning tasks rather than industrial thermal process control. Therefore, it remains unclear whether a Beta-based PPO policy can effectively reduce action oscillation and abrupt output changes in secondary cooling water systems.

To address the above issues, this paper proposes a Beta-policy and PID-inspired augmented-state proximal policy optimization (BPAS-PPO) framework for the secondary cooling water system. The proposed framework tailors state representation, bounded action parameterization, and reward design to the coupled dual-loop and actuator-constrained characteristics of the process. The main contributions are as follows:

(1) The development of a BPAS-PPO control structure enables simulation-level direct closed-loop control of the coupled dual-loop secondary cooling water system under actuator constraints.

(2) The design of the state representation incorporates PID-inspired error features to account for the coupling characteristics of the system, while a piecewise dense reward is constructed to support the dual-loop tracking task. Ablation studies further show that the fully PID-augmented state yields the most effective training representation among the tested alternatives.

(3) The validation of bounded policy parameterization is carried out through comparison with Gaussian-policy PPO under otherwise identical PPO settings. The results show that the Beta-policy design generates smoother control actions near actuator limits, while the complete BPAS-PPO framework achieves superior performance in step response, setpoint tracking, and disturbance rejection.

The remainder of this paper is organized as follows. In Section 2, the working principle and dynamic characteristics of the secondary cooling water system are introduced. In Section 3, the proposed BPAS-PPO method is presented in detail. In Section 4, the simulation setup and experimental results are presented. Finally, Section 5 concludes the paper and discusses future work.

## 2. Description of the Secondary Cooling Water System

### 2.1. Modeling of the Secondary Cooling Water System

**Figure 1:**
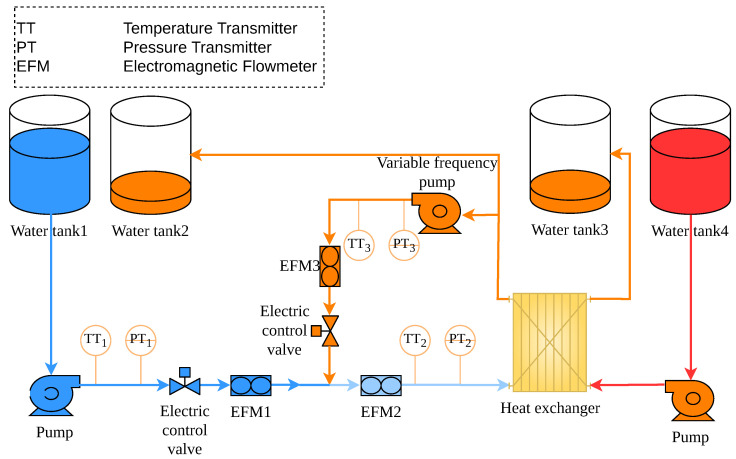
Process flow diagram of the secondary cooling water system.

The secondary cooling water system plays an important role in heat transfer and temperature regulation in industrial processes. As shown in Figure 1, it mainly comprises a heat exchanger, pumps, valves, electromagnetic flowmeters (0.3–8 m^3^/h, class 0.5), temperature transmitters (0–100 °C, 0.5% FS), and pressure transmitters (0–0.4 MPa, 0.5% FS).

The hydraulic circuit includes a cold-water branch and a return branch. In the cold-water branch, low-temperature water from Water Tank 1 is pumped into the main line. The branch flow rate is regulated by the electric control valve and measured by EFM1. In the return branch, a portion of the outlet water from the heat exchanger is recirculated to the mixing point by a variable-frequency pump, and the corresponding return flow rate is measured by EFM3. The mixed water then passes through EFM2 before entering the heat exchanger. TT1, PT1, TT2, PT2, TT3, and PT3 are installed at key locations to monitor the temperature and pressure conditions of the system. The inlet flow rate and inlet temperature of the heat exchanger are therefore determined by both the cold-water stream and the recirculated return stream.

The inlet conditions of the heat exchanger directly affect the heat-exchange load and are important for the stability of the heat-transfer process. For this reason, the inlet flow rate and inlet temperature of the heat exchanger are selected as the controlled variables in this study. They are denoted by $F_{\mathrm{in}}$ and $T_{\mathrm{in}}$, respectively. $F_{\mathrm{in}}$ is measured by EFM2, while $T_{\mathrm{in}}$ is measured by TT2. Let $F_{c}$ and $T_{c}$ denote the flow rate and temperature of the cold-water branch, respectively, and let $F_{r}$ and $T_{r}$ denote those of the recirculated return branch. Assuming mass conservation at the mixing point, complete mixing of the two streams, and negligible heat loss during the mixing process, the total inlet flow rate $F_{\mathrm{in}}$ and inlet temperature $T_{\mathrm{in}}$ of the heat exchanger can be expressed as

(1) $$F_{\mathrm{in}}=F_{c}+F_{r},$$

(2) $$T_{\mathrm{in}}=\frac{F_{c}T_{c}+F_{r}T_{r}}{F_{c}+F_{r}}.$$

Equations (1) and (2) show that changes in the branch flow rates directly affect the inlet flow rate $F_{\mathrm{in}}$. They also influence the inlet temperature $T_{\mathrm{in}}$ by changing the mixing ratio of the two streams. The valve opening and pump frequency therefore affect both controlled variables through their influence on $F_{c}$ and $F_{r}$. This indicates that the system exhibits non-negligible coupling characteristics. Equation (2) also shows that $T_{\mathrm{in}}$ is a nonlinear function of $F_{c}$ and $F_{r}$, which means that the physical process is inherently nonlinear.

**Figure 2:**
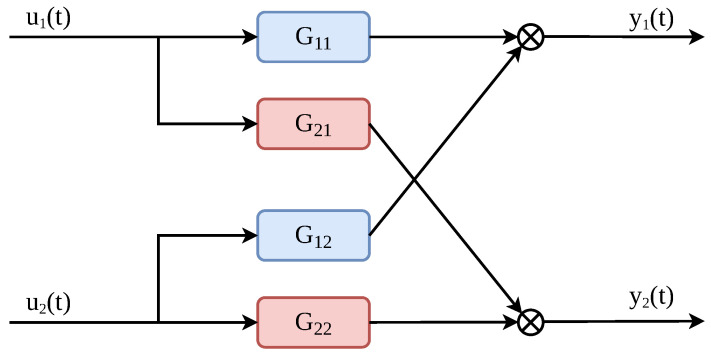
Two-input two-output structure of the secondary cooling water system.

Although the physical process exhibits nonlinear and coupled characteristics, for subsequent controller design and coupling analysis, the system is approximated within the selected operating region by a linear two-input two-output (TITO) model, whose input–output structure is shown in Figure 2. The manipulated variables are the valve opening in the cold-water branch, denoted by $u_{1}\left(t\right)$, and the pump frequency in the return branch, denoted by $u_{2}\left(t\right)$. The inlet flow rate and inlet temperature of the heat exchanger are taken as the controlled outputs, denoted by $y_{1}\left(t\right)$ and $y_{2}\left(t\right)$, respectively. Each transfer function $G_{ij}\left(s\right)$ describes the dynamic relationship from input $u_{j}\left(t\right)$ to output $y_{i}\left(t\right)$, where the first subscript denotes the output and the second denotes the input. For example, $G_{21}\left(s\right)$ represents the effect of the valve opening $u_{1}\left(t\right)$ on the inlet temperature $y_{2}\left(t\right)$.

In this study, system identification is carried out within a selected operating region, defined by the temperature interval [22, 35] °C and the flow-rate interval $\left[1.5,\;3.0\right]\;m^{3}/h$. This region is selected because the adopted linear TITO model structure provides relatively good identification quality within it. The operating condition $F_{\mathrm{in}}=2.3\;m^{3}/h$ and $T_{\mathrm{in}}=28$ °C is treated as a representative reference point within this region. Based on this input–output structure, step tests are conducted under operating conditions within the selected operating region, and the corresponding responses of $y_{1}\left(t\right)$ and $y_{2}\left(t\right)$ are recorded simultaneously. After data preprocessing, including denoising, detrending, time alignment, and outlier removal, a transfer function model is identified using the System Identification Toolbox in MATLAB R2023b. The identified transfer function matrix is given by

(3) $$G\left(s\right)=\left[\begin{array}{cc} \frac{0.05}{0.3s+1}e^{-0.3s} & \frac{0.06}{0.6s+1}e^{-0.6s}\\ \frac{-0.46}{0.3s+1}e^{-0.3s} & \frac{1.59}{0.6s+1}e^{-0.6s} \end{array}\right].$$

The identified model achieved fit values exceeding 85% for both the flow-rate and temperature outputs, and the residuals showed no obvious trend during identification. Therefore, the model in Equation (3) is used in this study as a bounded-region linear approximation for controller training and evaluation within the selected operating region, rather than as a full-range nonlinear description of the physical plant.

### 2.2. Coupling Characteristic Analysis of the Secondary Cooling Water System

To quantify the loop interaction and determine a reasonable input–output pairing, the relative gain array (RGA) is employed based on the steady-state gain matrix of the identified TITO model in Equation (3). By taking the zero-frequency limit, the steady-state gain matrix is obtained as

(4) $$K=\lim_{s\to 0}G\left(s\right)=\left[\begin{array}{cc} 0.05 & 0.06\\ -0.46 & 1.59 \end{array}\right].$$

The corresponding RGA matrix is calculated by

(5) $$\Lambda =K\circ {\left(K^{-1}\right)}^{T}=\left[\begin{array}{cc} 0.74 & 0.26\\ 0.26 & 0.74 \end{array}\right],$$

where ∘ denotes the Hadamard product.

For loop pairing analysis, diagonal RGA elements closer to unity are generally preferred because they imply weaker steady-state interaction. In this case, the diagonal elements are both 0.74, indicating that the coupling between the two loops is non-negligible. At the same time, the diagonal elements are larger than the off-diagonal ones, suggesting that pairing $u_{1}$ with $y_{1}$ and $u_{2}$ with $y_{2}$ is preferable to the off-diagonal pairing from the viewpoint of steady-state interaction. The RGA results confirm the coupling in the secondary cooling water system and support the selected input–output pairing.

## 3. Proposed BPAS-PPO Control Method

### 3.1. MDP Formulation of the Secondary Cooling Water System

The control problem of the secondary cooling water system is formulated as a Markov decision process (MDP), which is defined as

(6) M=(S,A,P,R,γ),

where *S* denotes the state space, *A* denotes the action space, *P* denotes the state transition probability, *R* denotes the reward function, and γ∈(0,1) is the discount factor. In deep reinforcement learning, the state transition dynamics are usually unknown to the agent, and the control policy is learned directly from interactions with the environment.

**Figure 3:**
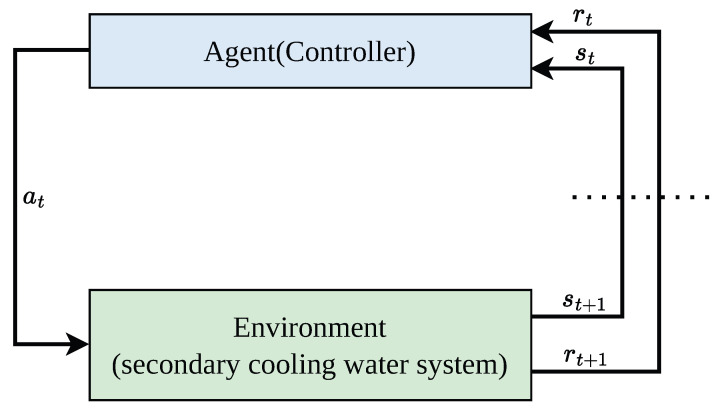
Reinforcement learning control framework for the secondary cooling water system.

In this study, the BPAS-PPO controller is regarded as the agent, while the secondary cooling water system is treated as the environment. As shown in Figure 3, at time step *t*, the agent observes the current state $s_{t}$ and outputs an action $a_{t}$, which is then applied to actuators such as the regulating valve and the variable-frequency pump. After the action is executed, the system evolves to the next state $s_{t+1}$ and returns a reward. Specifically, the state $s_{t}$ characterizes the current operating condition of the system as well as control-related error information, the action $a_{t}$ represents the control command generated by the controller, and the reward $r_{t}$ is used to evaluate the control effect and overall control performance. Under this formulation, the control objective is to learn an optimal policy that maximizes the expected cumulative reward.

### 3.2. State Design

In standard reinforcement learning frameworks, the agent typically relies on instantaneous observations as the state input. However, for the secondary cooling water system characterized by strong coupling and large thermal inertia, a single instantaneous observation is often insufficient to capture the dynamic evolution of the system as well as the accumulated historical deviation. Such a limited state representation may restrict the learning capability of the agent and slow down policy convergence during training. Therefore, inspired by the temporal error decomposition mechanism in classical PID control, the state representation is reconstructed to incorporate richer dynamic information.

For the *i*-th controlled variable (i∈{1,2}, where i=1 represents the inlet flow rate $F_{\mathrm{in}}$ and i=2 represents the inlet temperature $T_{\mathrm{in}}$), let $sp_{i,t}$ and $y_{i,t}$ denote the control setpoint and system output at time step *t*, respectively. Three types of error features are extracted to construct the augmented state representation, including the tracking error, the integral error, and the derivative error:

(7) $$e_{i,t}=sp_{i,t}-y_{i,t};\;e_{i,t}^{I}=\sum_{\tau =0}^{t}e_{i,\tau }\;\Delta t;\;e_{i,t}^{D}=\frac{e_{i,t}-e_{i,t-1}}{\Delta t},$$

where $e_{i,t}$, $e_{i,t}^{I}$, and $e_{i,t}^{D}$ denote the tracking error, integral error, and derivative error of the *i*-th controlled variable, respectively.

Based on these variables, four progressively augmented state representations are constructed as follows:

(8) $$\begin{array}{cc} S_{P} & ={\left[sp_{1,t},y_{1,t},e_{1,t},sp_{2,t},y_{2,t},e_{2,t}\right]}^{⊤},\\ S_{PI} & ={\left[{\left(S_{P}\right)}^{⊤},e_{1,t}^{I},e_{2,t}^{I}\right]}^{⊤},\\ S_{PD} & ={\left[{\left(S_{P}\right)}^{⊤},e_{1,t}^{D},e_{2,t}^{D}\right]}^{⊤},\\ S_{PID} & ={\left[{\left(S_{P}\right)}^{⊤},e_{1,t}^{I},e_{1,t}^{D},e_{2,t}^{I},e_{2,t}^{D}\right]}^{⊤}, \end{array}$$

where $S_{P}$, $S_{PI}$, $S_{PD}$, and $S_{PID}$ denote the P-type, PI-type, PD-type, and PID-type state representations, respectively. Accordingly, $S_{P}\in R^{6}$, $S_{PI},S_{PD}\in R^{8}$, and $S_{PID}\in R^{10}$. These state formulations are employed in the ablation study to evaluate the contribution of different error components to policy learning.

### 3.3. Action Design

The action $a_{t}$ denotes the control input selected by the agent when the system is in state $s_{t}$. For the secondary cooling water system considered in this study, the action components correspond to the manipulated variables of two actuators, namely, the opening of the electric regulating valve and the operating frequency of the variable-frequency pump. Therefore, at time step *t*, the action vector is defined as

(9) $$A={\left[u_{1,t},\;u_{2,t}\right]}^{⊤},$$

where $u_{1,t}$ denotes the valve opening in the range of [0,100]%, and $u_{2,t}$ denotes the pump operating frequency in the range of [0,60]Hz.

Since the learned actions are implemented through physical actuators, such as electric regulating valves and variable-frequency pumps, their magnitudes must remain within the admissible action ranges. Therefore, action magnitude is considered an important engineering criterion in controller evaluation.

### 3.4. Reward Design

The reward function should provide clear feedback for tracking performance and support stable policy learning. For the secondary cooling water system, temperature and flow rate are strongly coupled. Under this condition, a sparse reward often provides limited guidance, especially in the early stage of training. To address this issue, a piecewise dense reward function is designed based on the tracking error.

For the *i*-th control loop, the tracking error at time step *t* is denoted by $e_{i,t}$. The reward of each loop is defined as

(10) $$r_{i,t}=\left\{\begin{array}{cc} \omega_{i}\left(\rho_{i}-\exp\left(k_{i}\left|e_{i,t}\right|\right)\right), & |e_{i,t}|\;<\;\eta_{i}\\ -\mu_{i}\left|e_{i,t}\right|, & |e_{i,t}|\;\geq \;\eta_{i} \end{array}\right.,$$

where $\eta_{i}$ is the predefined acceptable error bound, $\mu_{i}$ is the linear penalty weight, $\omega_{i}$ is the basic reward gain, $\rho_{i}$ is the offset constant, and $k_{i}$ is the error sensitivity coefficient.

This reward structure has two regions. When the tracking error is within the acceptable bound, an exponential-form reward is used to provide refined guidance near the setpoint. This helps the agent distinguish small performance differences and improves tracking accuracy. When the tracking error exceeds the acceptable bound, a linear penalty is applied to strengthen the correction effect and encourage the agent to reduce the deviation quickly.

The total reward of the secondary cooling water system is defined as the sum of the rewards from the two control loops:

(11) $$R=r_{1,t}+r_{2,t}.$$

In this study, the reward parameters are set as follows. The thresholds $\eta_{1}=\eta_{2}=0.1$ define the acceptable steady-state deviation around the setpoint. The offsets $\rho_{1}=\rho_{2}=2$ ensure a positive reward signal in the vicinity of the target state. The coefficients $k_{1}=k_{2}=6.93$ were chosen so that the exponential reward term approaches zero near the switching threshold |e|=η, thereby producing a numerically smooth transition between the two reward regions. The scaling weights $\omega_{1}=\omega_{2}=10$ were determined empirically, as smaller values produced insufficient gradient signals while larger values tended to destabilize training. The loop weights $\mu_{1}=0.5$ and $\mu_{2}=1$ reflect the greater practical importance of temperature regulation in the secondary cooling water system, while also promoting balanced convergence across both control loops during training.

### 3.5. Beta-Distribution-Based Policy Improvement for Continuous Action Spaces

In continuous-control problems, PPO commonly employs a Gaussian policy to sample actions. However, the Gaussian distribution has unbounded support over (−∞,+∞), which is inconsistent with the bounded action requirements of practical control systems. In the secondary cooling water system considered in this study, both manipulated variables are subject to strict physical constraints. To enable bounded policy modeling, an intermediate normalized action variable is introduced over the interval [0,1].

When a Gaussian policy is used in a bounded action space, sampled actions that exceed the admissible action range are typically projected back by a clipping operation,

(12) $${\tilde{x}}_{t}=\mathrm{clip}\left(x_{t},0,1\right),$$

where $x_{t}$ denotes the sampled normalized action. However, PPO updates the policy according to the log-probability of the sampled action $x_{t}$, whereas the environment evolves according to the clipped action ${\tilde{x}}_{t}$. This mismatch may reduce the consistency of policy optimization. In addition, actions sampled near or beyond the boundaries may accumulate at the clipping points. This may increase action saturation and degrade control smoothness.

To alleviate this issue, the Gaussian policy is replaced by a Beta distribution, whose support is naturally bounded within [0,1]. Therefore, the sampled normalized actions directly satisfy the admissible action range without requiring any additional clipping operation. For the *i*-th action dimension, the probability density function of the Beta distribution is defined as

(13) $$f\left(x_{i,t};\alpha_{i,t},\beta_{i,t}\right)=\frac{1}{B(\alpha_{i,t},\beta_{i,t})}x_{i,t}^{\alpha_{i,t}-1}{\left(1-x_{i,t}\right)}^{\beta_{i,t}-1},\;x_{i,t}\in \left[0,1\right],$$

where $\alpha_{i,t}>0$ and $\beta_{i,t}>0$ are the shape parameters, and the Beta function is

(14) $$B\left(\alpha_{i,t},\beta_{i,t}\right)=\frac{\Gamma \left(\alpha_{i,t}\right)\Gamma \left(\beta_{i,t}\right)}{\Gamma (\alpha_{i,t}+\beta_{i,t})}.$$

Let the normalized action vector be denoted by

(15) $$x_{t}={\left[x_{1,t},x_{2,t}\right]}^{⊤}\in {\left[0,1\right]}^{2}.$$

Assuming conditional independence between action dimensions, the joint policy is factorized as

(16) $$\pi_{\theta }\left(x_{t}|s_{t}\right)=\prod_{i=1}^{2}f\left(x_{i,t};\alpha_{i,t},\beta_{i,t}\right).$$

To ensure valid Beta-distribution parameters, the policy-network outputs corresponding to $\alpha_{i,t}$ and $\beta_{i,t}$ are transformed into positive values. In this way, the original PPO optimization framework remains unchanged, while only the action distribution parameterization is replaced.

The normalized actions are then mapped to the physical control inputs defined in Section 3.3:

(17) $$u_{i,t}=u_{i,\min}+x_{i,t}\left(u_{i,\max}-u_{i,\min}\right),$$

where $u_{i,\min}$ and $u_{i,\max}$ denote the lower and upper bounds of the *i*-th actuator, respectively. In this study, the valve opening is limited to [0,100]%, and the pump frequency is limited to [0,60]Hz. Therefore, the corresponding mappings are expressed as

(18) $$u_{1,t}=100x_{1,t},\;u_{2,t}=60x_{2,t}.$$

With this design, the generated control inputs always satisfy the actuator constraints, which is beneficial for improving the stability and smoothness of policy learning in bounded continuous-action control tasks.

### 3.6. Network Architecture and Algorithm Design

PPO is an improved actor–critic reinforcement learning algorithm. Within this framework, the decision-making process is jointly carried out by an actor network and a critic network. The actor network interacts with the environment and outputs actions based on the current observed state, whereas the critic network evaluates the quality of the action selected by the actor under the current state and provides guidance for policy improvement toward higher long-term expected rewards. On this basis, PPO further introduces a clipped surrogate objective, which effectively constrains the update magnitude of the policy and enhances training stability.

In this study, both the actor and critic networks take as input the PID-augmented state representation $S_{PID}\in R^{10}$ defined in Section 3.2. Each network consists of two fully connected hidden layers with 64 and 32 neurons. The actor and critic are trained independently and do not share hidden-layer parameters.

At the output stage, the actor network does not adopt a conventional Gaussian policy. Instead, it employs the Beta-distribution-based policy structure introduced in Section 3.5. For each action dimension, the actor outputs two shape parameters, denoted by $\alpha_{i,t}$ and $\beta_{i,t}$. To ensure that the distribution parameters remain strictly positive, the output layer uses the Softplus activation function. In addition, a bias term is introduced to further constrain the parameter range, thereby improving distribution regularity, monotonicity, and numerical stability. Meanwhile, the critic network uses a linear output neuron to directly estimate the state value function $V(s_{t})$, which is used for advantage estimation and policy optimization.

For the multivariable setpoint tracking task of the secondary cooling water system, random initialization of the control setpoints is performed at the beginning of each training episode within the physically admissible operating range of the system. Specifically, the admissible operating region D is defined as the temperature setpoint interval [22,35] °C and the flow-rate setpoint interval $\left[1.5,3.0\right]\;m^{3}/h$. This randomization strategy exposes the agent to diverse setpoint conditions throughout the training region, reducing sensitivity to any single fixed target and improving policy coverage across the training domain. The complete execution procedure of the BPAS-PPO algorithm is summarized in Algorithm 1.
**Algorithm 1** Training procedure of BPAS-PPO** Input:** Admissible operating region D and hyperparameters $\gamma ,\lambda ,\epsilon ,M_{\pi },M_{V},T,K$**Output:** Trained policy parameters θ  1:Randomly initialize actor parameters θ and critic parameters ϕ  2:Set $\theta_{\mathrm{old}}\leftarrow \theta$  3:**for** k=1,2,…,K **do**  4:      Randomly sample a setpoint vector ${\mathrm{SP}}_{k}$ from D  5:      Initialize an empty trajectory $D_{k}$  6:      **for** t=1,2,…,T **do**  7:            Construct the PID-augmented state $s_{t}^{\mathrm{PID}}$  8:            Compute the Beta-policy parameters from the actor network  9:            Sample normalized action $x_{t}∼\pi_{\theta_{\mathrm{old}}}\left(\cdot |s_{t}^{\mathrm{PID}}\right)$10:            Map $x_{t}$ to the physical control input $u_{t}$ using Equation (18)11:            Execute $u_{t}$ and observe reward $r_{t}$ and next state $s_{t+1}^{\mathrm{PID}}$12:            Store transition $\left(s_{t}^{\mathrm{PID}},x_{t},r_{t},s_{t+1}^{\mathrm{PID}}\right)$ in $D_{k}$13:      **end for**14:      Compute value targets ${\hat{R}}_{t}$ and generalized advantage estimates ${\hat{A}}_{t}$ from $D_{k}$15:      Normalize ${\hat{A}}_{t}$ over the trajectory16:      **for** $m=1,2,\ldots ,M_{\pi }$ **do**17:            Update actor parameters θ by maximizing the clipped PPO objective18:      **end for**19:      **for** $m=1,2,\ldots ,M_{V}$ **do**20:            Update critic parameters ϕ by minimizing the value loss21:      **end for**22:      Set $\theta_{\mathrm{old}}\leftarrow \theta$23:**end for**24:**return** θ

## 4. Experiment and Result Analysis

**Figure 4:**
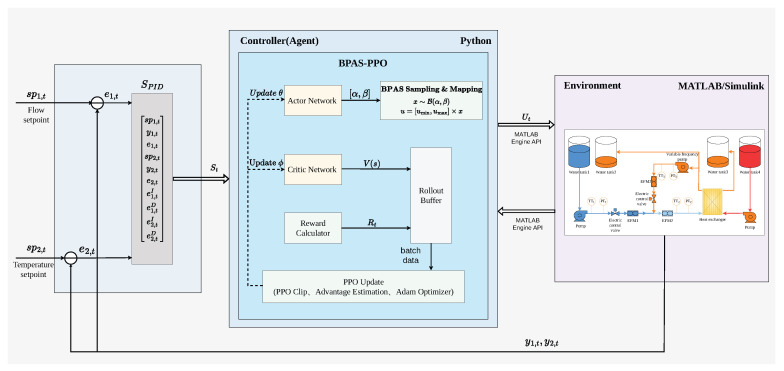
Framework of the proposed co-simulation system.

To evaluate the control performance of the proposed BPAS-PPO algorithm in the secondary cooling water system, a co-simulation platform based on Python 3.9 and MATLAB R2023b was developed. Specifically, the simulation environment of the secondary cooling water system was constructed in Simulink using the transfer-function matrix model derived in Section 2.1. Data interaction between MATLAB and Python was achieved through the MATLAB Engine API for Python, thereby enabling policy training. The overall architecture of the co-simulation platform is shown in Figure 4.

Considering that practical industrial environments are commonly affected by external disturbances, random disturbance signals were injected into the control input channel of the simulation environment to enhance the robustness of the controller. The main parameters used in agent training are listed in Table 1. All computations were performed on a laptop (Dell Inc., Round Rock, TX, USA) equipped with Windows 11 (Microsoft Corp., Redmond, WA, USA), an AMD Ryzen 7 5800H CPU @ 3.20 GHz (Advanced Micro Devices, Inc., Santa Clara, CA, USA), 16 GB RAM, and an NVIDIA RTX 3060 GPU (NVIDIA Corp., Santa Clara, CA, USA).

For real-time control systems, the control cycle is a critical design parameter. To assess the computational feasibility of the proposed BPAS-PPO controller, the offline training time and the per-step online inference time were evaluated and are summarized in Table 2.

As shown in Table 2, the mean per-step inference time is 0.53 ms, corresponding to only 0.53% of the 100 ms sampling period. The maximum inference time is 1.64 ms, which remains well within the control cycle. These results indicate that the proposed BPAS-PPO controller satisfies the real-time requirements of the current simulation framework.

### 4.1. Convergence Analysis of Agent Training

**Figure 5:**
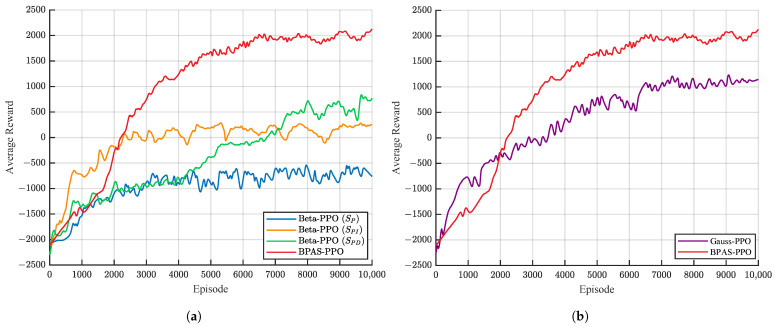
Average reward curves during training: (**a**) Average reward curves of the Beta-distribution policy networks under different state-space configurations; (**b**) Average reward curves of Gauss-PPO and BPAS-PPO under the complete PID-augmented state space.

To evaluate the effectiveness of the proposed state-space-guided Beta-distribution policy network, five PPO-based methods were compared using their average reward curves during training, including Beta-PPO ($S_{P}$), Beta-PPO ($S_{PI}$), Beta-PPO ($S_{PD}$), Gauss-PPO, and BPAS-PPO. Each method was trained five times with different random seeds, and the mean reward over the five runs was taken as the final result. All methods used the same simulation environment, network architecture, and core hyperparameter settings. A moving average with a window size of 50 was applied to smooth the reward curves and improve their readability. The smoothed average training reward curves are shown in Figure 5.

As shown in Figure 5a, the state-space configuration has a strong impact on learning performance. BPAS-PPO with the complete PID state achieves the fastest convergence and the highest final reward. Its training process becomes stable after about 6000 episodes. Beta-PPO ($S_{PD}$), which excludes the integral term, shows a rapid reward increase in the early stage. However, it cannot remove the steady-state error, leading to limited final performance. This result demonstrates the role of the integral term in preserving historical error information and compensating for continuous disturbances. Beta-PPO ($S_{PI}$), without the differential term, converges much more slowly. Beta-PPO ($S_{P}$), which uses only the position error, remains in the negative reward region during the whole training process. This suggests that a single error signal cannot provide enough state information for effective policy learning. As shown in Figure 5b, Gauss-PPO exhibits stronger reward oscillations and lower final reward. In contrast, BPAS-PPO converges faster and performs better at convergence.

According to the training results, BPAS-PPO and Gauss-PPO are chosen as the reinforcement learning methods for the subsequent control performance experiments. Gauss-PPO serves as a direct baseline that shares the exact same PPO framework, state representation, reward function, and training settings as BPAS-PPO, differing only in the policy distribution. This controlled setup specifically isolates the performance impact of the Beta-distribution policy against conventional Gaussian clipping, rather than providing an exhaustive benchmark of all bounded-action DRL algorithms. Furthermore, classical PID and Fuzzy-PID are introduced as conventional benchmark controllers. For a fair comparison, both were implemented under the same plant model and sampling period. The PID baseline was initialized via the Ziegler–Nichols method [32] and fine-tuned for transient response, whereas the Fuzzy-PID employed a standard self-tuning framework based on tracking errors [33].

### 4.2. Step Response and Control Performance Simulation

**Figure 6:**
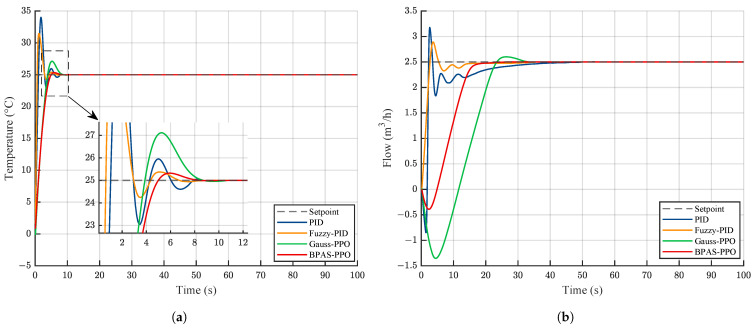
Comparison of step response performance under different control methods: (**a**) Temperature loop; (**b**) Flow loop.

To verify the control performance of BPAS-PPO in the secondary cooling water system, a 100 s simulation experiment was conducted. The temperature setpoint was set to 25 °C, and the flow-rate setpoint was set to $2.5\;m^{3}/h$. The control responses of different methods are shown in Figure 6.

As shown in Figure 6, compared with the other control methods, the proposed BPAS-PPO achieves better performance in terms of settling time and overshoot. This indicates that BPAS-PPO can better balance tracking accuracy, response speed, and system stability. In contrast, Gauss-PPO exhibits larger overshoot and longer settling time. The performance indices of different control methods for the temperature and flow-rate loops are summarized in Table 3.

The results in Table 3 should be assessed with reference to the practical relevance of the transient indices. Rise time only characterizes the initial response speed and does not, by itself, determine control performance. In the secondary cooling water system, overshoot and settling time are more important because they reflect how quickly the process can reach a stable operating condition without pronounced oscillation. On this basis, BPAS-PPO provides the most balanced transient response among the compared methods. In the temperature loop, despite a longer rise time, it limits overshoot to 1.29% and achieves the shortest settling time of 3.91 s. A similar pattern is observed in the flow loop, where it again gives the smallest overshoot and the shortest settling time. The slower rise of BPAS-PPO is therefore outweighed by its superior settling behavior and overall regulation performance.

**Figure 7:**
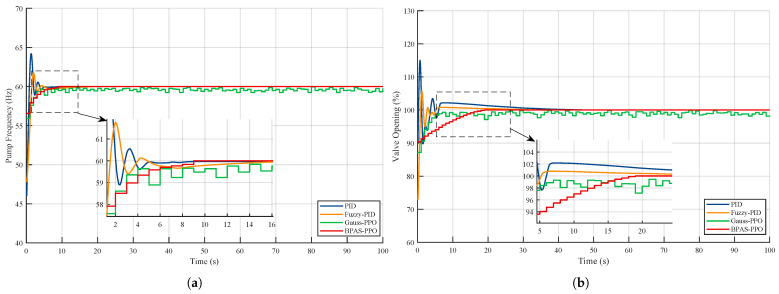
Control signal comparison under different control methods: (**a**) Pump frequency; (**b**) Valve opening.

In addition, to further analyze the internal mechanism by which the Beta-distribution policy improves performance, a comparative study of the control signals was conducted under extreme operating conditions close to the physical limits of the actuator. Under such conditions, the control input tends to approach its constrained boundaries during the transient process, making the behavioral differences among different policy distributions more evident. Figure 7 shows the dynamic variations of the control signals generated by different methods when the temperature setpoint changes abruptly from 25 °C to 35 °C and the flow rate increases from $2.5\;m^{3}/h$ to $3\;m^{3}/h$.

As shown in Figure 7, PID and Fuzzy-PID exhibit evident control saturation in the initial stage, which may induce integral windup and degrade transient performance. In addition, when Gauss-PPO approaches the control boundaries, the clipping operation introduces high-frequency oscillations. This not only degrades control performance but also increases actuator wear. In contrast, BPAS-PPO naturally generates smooth and bounded control signals without the need for clipping, owing to the inherent bounded-support property of the Beta distribution. This property ensures fast and overshoot-free tracking while maintaining smooth control actions throughout the entire operating range, thereby enhancing actuator longevity and operational reliability.

### 4.3. Tracking Performance Simulation Experiments

**Figure 8:**
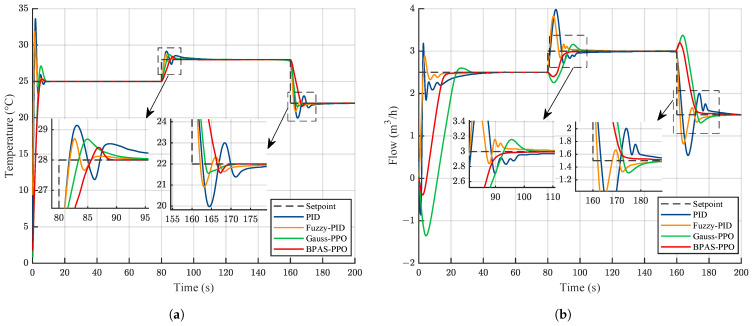
Comparison of tracking performance under different control methods: (**a**) Temperature loop; (**b**) Flow loop.

To evaluate the tracking performance of BPAS-PPO, time-varying setpoints were designed for both the temperature and flow control loops. During the 200 s simulation, the temperature setpoint was changed to 25 °C, 28 °C, and 22 °C at 0 s, 80 s, and 160 s, respectively. Meanwhile, the flow setpoint was set to $2.5\;m^{3}/h$, $3\;m^{3}/h$, and $1.5\;m^{3}/h$ at the same switching instants. The tracking responses of different control methods are shown in Figure 8.

As shown in Figure 8a, in the temperature control loop, both conventional PID and Fuzzy-PID exhibit obvious overshoot and oscillation when the setpoint changes. In contrast, Gauss-PPO and BPAS-PPO show significantly better tracking performance than the other methods. A closer view further indicates that BPAS-PPO has a shorter settling time than Gauss-PPO. In addition, when the setpoint changes, the flow control loop is disturbed by the temperature loop, resulting in noticeable differences in the transient responses of different controllers, as shown in Figure 8b. Conventional PID and Fuzzy-PID cannot effectively suppress this coupling disturbance and require a relatively long time to reach the steady state. Although Gauss-PPO responds quickly, it still needs a longer adjustment process before becoming stable. Overall, BPAS-PPO achieves satisfactory control performance in both control loops.

### 4.4. Robustness Performance Simulation Experiments

**Figure 9:**
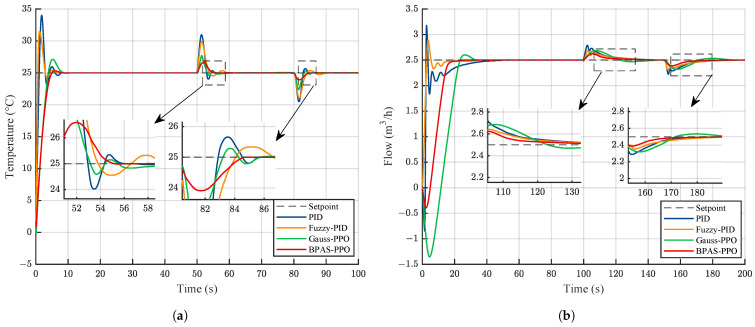
Comparison of disturbance rejection performance under different control methods: (**a**) Temperature loop; (**b**) Flow loop.

To evaluate the disturbance rejection performance of BPAS-PPO under external disturbances, step disturbance signals were injected into the control ports of the system. In the temperature-loop test, a 100 s simulation was conducted, and step disturbances of +5 Hz and −5 Hz were applied to the pump-frequency channel $u_{2}$ at 50 s and 80 s, respectively. Similarly, in the flow-loop test, a 200 s simulation was performed, and step disturbances of +5% and −5% were applied to the valve-opening channel $u_{1}$ at 100 s and 150 s, respectively. The experimental results are shown in Figure 9.

The experimental results indicate that both the conventional PID and Fuzzy-PID controllers exhibit dynamic deviations and decaying oscillations under external disturbances. Although Gauss-PPO shows a relatively fast response, it still requires more time than BPAS-PPO to reach the steady state. In contrast, BPAS-PPO enables the system to return to the setpoint most rapidly. Compared with the other methods, it exhibits the best disturbance rejection performance and robustness.

**Figure 10:**
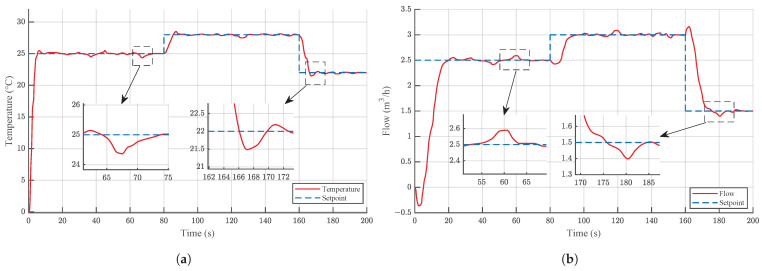
Setpoint tracking performance of BPAS-PPO under sensor noise (σ=0.1): (**a**) Temperature loop; (**b**) Flow loop.

Additionally, to further evaluate the robustness of BPAS-PPO under sensor noise, Gaussian white noise with zero mean and standard deviation σ=0.1 was added independently to both measurement channels ($y_{1}$ and $y_{2}$) during the setpoint-tracking simulation described in Section 4.3. The tracking responses are shown in Figure 10.

As shown in Figure 10, BPAS-PPO achieves relatively stable setpoint tracking throughout the 200 s simulation under continuous Gaussian sensor noise with σ=0.1. The enlarged views reveal localized deviations at specific instants. In the temperature loop, deviations of approximately 0.5 °C are observed near 67 s and 167 s. In the flow loop, deviations of approximately $0.1\;m^{3}/h$ are observed near 60 s and 180 s. Despite these localized deviations, the output recovers to each setpoint without sustained oscillation, demonstrating acceptable robustness of BPAS-PPO against measurement noise.

## 5. Conclusions

This paper develops a Beta-policy and PID-inspired augmented-state proximal policy optimization (BPAS-PPO) framework for the secondary cooling water system. The proposed framework enables simulation-level direct closed-loop control of the coupled dual-loop task under actuator constraints. The state representation, bounded action parameterization, and reward design are tailored to the characteristics of the process. Ablation experiments show that the complete PID-augmented state provides the most effective training representation among the tested alternatives. Comparative simulations within the selected operating region further show that BPAS-PPO achieves better overall control performance than PID, Fuzzy-PID, and Gauss-PPO in terms of overshoot suppression, settling behavior, setpoint tracking, disturbance rejection, and control smoothness near actuator limits. These results demonstrate the practical value of the proposed framework for the studied coupled and actuator-constrained thermal process.

The results indicate that BPAS-PPO can provide effective control of the secondary cooling water system within the selected operating region considered in this study. The design of the state representation, bounded policy parameterization, and reward function may also provide useful insights for other coupled and actuator-constrained thermal process control problems. At the same time, the present study remains subject to several limitations. The current validation is conducted in a co-simulation environment and relies on an identified linear TITO model used as a bounded-region approximation of the plant dynamics. The reported results should therefore be interpreted as evidence of effectiveness within the selected operating region, rather than across the full nonlinear operating range of the physical plant. In addition, the robustness assessment and the DRL benchmark comparison remain limited in scope. The generalization ability, safety, and interpretability of the learned policy therefore still require further investigation.

Future work will focus on higher-fidelity validation and broader performance assessment of the proposed framework. A more detailed physical model of the secondary cooling water system will be developed, including heat-exchange dynamics, hydraulic nonlinearities, and nonlinear actuator characteristics of valves and pumps. On this basis, the proposed framework will be evaluated beyond the selected operating region to provide stronger evidence of generalization under broader operating conditions. More comprehensive robustness studies will also be conducted under more complex sensor-noise conditions and other realistic industrial uncertainties. In parallel, broader comparisons with squashed-Gaussian PPO variants and other advanced bounded-action deep reinforcement learning methods will be included. Ultimately, a real physical experimental platform will be built to validate the actual control performance of the proposed framework in practical applications.

## Figures and Tables

**Table 1:** Parameter settings of the proposed BPAS-PPO algorithm.

Hyperparameter	Setting
Sampling time	0.1 s
Simulation time per episode	20 s
Number of training episodes *K*	10,000
Trajectory length *T*	200 steps
Discount factor γ	0.98
GAE parameter λ	0.95
PPO clipping ratio ϵ	0.2
Actor update epochs $M_{\pi }$	10
Critic update epochs $M_{V}$	10
Critic hidden layers	64, 32
Shared actor hidden layers	64, 32
Alpha head hidden layer	32
Beta head hidden layer	32
Critic learning rate	0.001
Actor learning rate	0.0005
Mini-batch size	128

**Table 2:** Computational burden of the proposed BPAS-PPO controller.

Metric	Value
Offline training time	6∼7 h
Number of inference runs	10,000
Mean inference time	0.53 ms
Maximum inference time	1.64 ms
Minimum inference time	0.48 ms
Standard deviation	0.06 ms
Sampling period	100 ms
Mean/Sampling period	0.53%

**Table 3:** Performance indices of different control methods.

Loop	Control Method	Rise Time (s)	Settling Time (s)	Overshoot (%)
Temperature Loop	PID	0.82	7.41	36.05
	Fuzzy-PID	0.46	5.84	25.88
	Gauss-PPO	2.88	7.80	8.46
	**BPAS-PPO**	**3.35**	**3.91**	**1.29**
Flow Loop	PID	0.34	38.57	27.09
	Fuzzy-PID	1.97	28.32	15.62
	Gauss-PPO	8.93	31.43	4.04
	**BPAS-PPO**	**8.37**	**19.23**	**0.12**

**Bold** indicates the proposed method (BPAS-PPO) and its performance values.

## Data Availability

The original contributions presented in this study are included in the article. Further inquiries can be directed to the corresponding author.
